# A wearable telehealth system for the monitoring of parameters related to heart failure

**DOI:** 10.1016/j.heliyon.2024.e26841

**Published:** 2024-02-22

**Authors:** Sheikh MA. Iqbal, Mary Ann Leavitt, Guerline Pedilus, Imadeldin Mahgoub, Waseem Asghar

**Affiliations:** a Department of Electrical Engineering & Computer Science, Florida Atlantic University, Boca Raton, FL, 33431, USA; b Asghar-Lab, Micro and Nanotechnology in Medicine, College of Engineering and Computer Science, Boca Raton, FL, 33431, USA; c Christine E. Lynn College of Nursing, Florida Atlantic University, Boca Raton, FL, 33431, USA; d Department of Biological Sciences (Courtesy appointment), Florida Atlantic University, Boca Raton, FL, 33431, USA

## Abstract

Heart failure is a cardiovascular disease in which heart fails to pump sufficient blood required by the body. Significant signs of worsening heart failure include decreased thoracic impedance, increased heart rate, irregular electrocardiogram (ECG), and lack of motion activity of the patient. Heart failure can be better managed if monitored continuously and in real-time. The existing solutions for continuous monitoring of these parameters are invasive and hence are not only expensive but can also cause serious health risks. This paper discusses the development of a telehealth system that consists of an Internet of Things including a wearable device connected to a cloud-based database and a mobile application using Wi-Fi. The wearable device is a noninvasive monitor that consists of different sensors embedded with a microcontroller and can be a potential solution for better management of heart failure. It continuously monitors the above-mentioned parameters and sends data to the mobile application using a cloud-based system. The mobile application has separate portals for patients and doctors where doctor can monitor a specific patient enrolled under his profile. The performance of the developed device is validated in 10 healthy individuals.

## 1 Introduction

Heart failure (HF) is a cardiovascular disease affecting 64 million people around the world. In United States (US), more than 6 million people over the age of 20 years are diagnosed with HF (US) [1]. According to American Heart Association, HF is projected to increase by 46% with close to 8 million HF patients in 2030 [2]. HF is a structural problem in which the heart is unable to pump sufficient blood to meet the requirements of the body. A healthy heart pumps 55%–60% of the volume of the left ventricle. However, in HF, the heart pumps less than this amount. HF can be categorized into two types: systolic and diastolic HF [3]. In systolic HF, the heart pumps less than or equal to 40% of the total blood inside the left ventricle. Systolic HF is also called HF with reduced ejection fraction (HFrEF). In diastolic HF, the ventricle is thickened and stiff and holds a lesser volume of blood. Although the heart has a normal ejection fraction of 50% or more, the volume pumped with each heartbeat is less than normal. Diastolic HF is also known as HF with preserved ejection fraction (HFpEF) [3]. Both types of HF reduce the cardiac output of blood and oxygen, causing symptoms of fatigue, shortness of breath, and fluid retention.

Currently, the commercially available solutions for monitoring HF are invasive and primarily for HFrEF. To the best of our knowledge, there are no non-invasive solution for patients with HFpEF which is 50% of HF patients. Currently available solutions include Implantable Cardioverter Defibrillator (ICD) and CardioMEMS [4,5]. Patients with HFrEF are eligible for implantable cardioverter defibrillator (ICD). An ICD is an implantable device that monitors physiological parameters related to HF [[6], [7], [8], [9]]. These parameters include heart rate, intrathoracic impedance, ECG, activity status, and heart rate variability [10,11]. An ICD implant costs around $37,000 [4]. Patients with HFrEF or HFpEF can receive a CardioMEMS™ device [5]. CardioMEMS is also an implantable device that measures pulmonary artery pressure to monitor the progression of HF. Both solutions are not only expensive but also have surgical risks [5]. Most of the commercially available devices are not in accordance with the World Health Organization's (WHO) ASSURED criteria for Affordable, Sensitive, Specific, User-friendly, Rapid and robust, Equipment-free and Deliverable devices [[12], [13], [14]]. Therefore, there is a need for a non-invasive solution that can provide continuous and real-time monitoring of parameters related to HF. Wearable devices along with telehealth monitoring can provide Point of Care (POC) diagnostic for continuous and real-time monitoring of the vital parameters of HF [15,16].

A telehealth system allows the medical provider real-time visualization of vital parameters so they can make timely treatment decisions [17]. As HF is a chronic disease with symptoms that may not be evident in early stages, it is essential to have a telehealth monitoring system for parameters indicating worsening HF. A system that can integrate the non-invasive device with the internet, as part of the internet of things (IoT), will allow the medical practitioner access to vital patient information.

We have previously developed one such device that continuously monitors parameters significant to HF [18]. These parameters include transthoracic impedance, ECG, heart rate, and activity status [11,18]. These are similar to parameters monitored in the standard ICD. Thoracic impedance is the electrical impedance within the thorax region of the human body and is an early indicator of the HF [19]. A reduction in the thoracic impedance indicates increased fluid retention which can be an early sign of worsening HF [[20], [21], [22]]. This is because when cardiac output decreases, fluid starts to accumulate in the thoracic region due to reduced heart performance. This fluid retention decreases the thoracic impedance in that area. Another symptom of HF is the reduced activity of the patient that might be due to several factors: shortness of breath from decreased cardiac output or swelling in the legs. Moreover, to compensate for the decreased cardiac output the heart pumps at a faster rate which increases the heart rate of the HF patient. Similarly, ECG is a standard diagnostic bio signal significant for cardiovascular disease and is important in the assessment of the heart's performance. In this paper, we discuss a telehealth monitoring system that includes the integration of the improved version of the previously developed device along with a Wi-Fi-enabled mobile application. The mobile application has separate portals for doctors and patients where doctors can monitor the above-mentioned parameters of their enrolled patients continuously and in real-time.

The subsequent paragraphs will discuss the development of the device and the mobile application. We have tested the device on a diverse set of healthy individuals in different real-life conditions and their parameters were monitored in real-time. Their results will also be discussed in the following sections.

## 2 Methodology

**Fig. 1:**
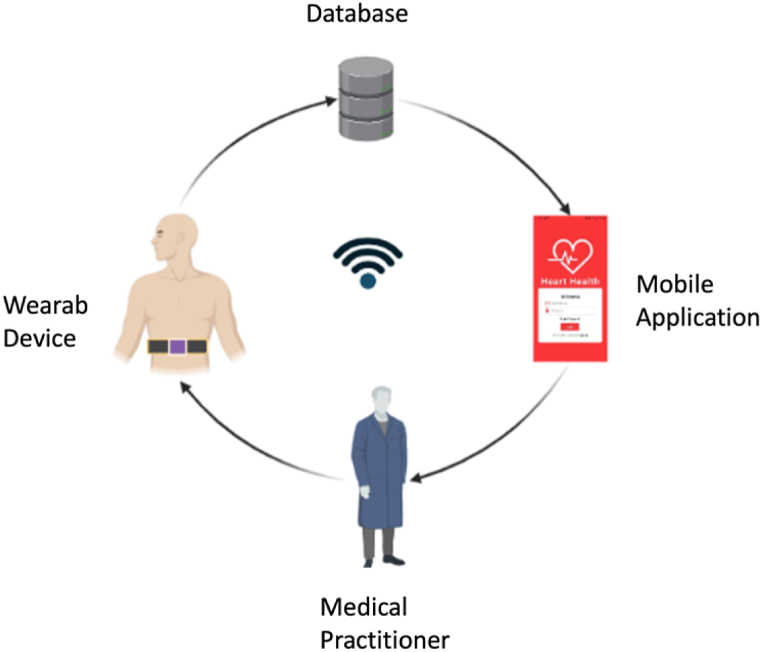
Flow Diagram of the telehealth monitoring system.

The telehealth system designed consists of the wearable device, the cloud-based database, and the mobile application, as shown in Fig. 1. The wearable device embeds different sensors with the microcontroller and connects the device with the cloud server using Wi-Fi. The cloud database is connected to the mobile application where the parameters recorded with the wearable device are monitored in real-time. The detail of these modules is explained in the subsequent sections.

### 2.1 Wearable device

**Fig. 2:**
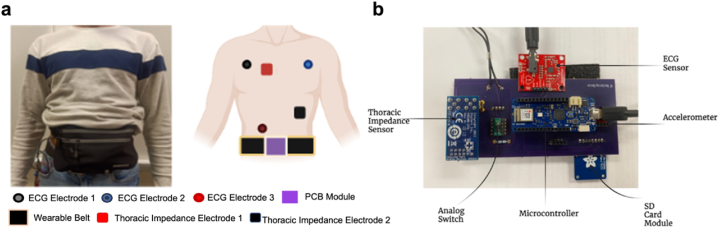
The healthcare wearable belt a. The healthcare wearable belt along with the placement of electrodes on the subject b. The healthcare wearable device module along with different sensors embedded with the microcontroller.

The previously designed healthcare wearable device has been improved to enable telehealth monitoring. For this purpose, a microcontroller with a smaller size and a built-in Wi-Fi module has been used. Arduino MKR 1010 is a suitable microcontroller for most of the IoT applications [23]. The microcontroller enables all the functionality as the previously used microcontroller with the additional functionality of Wi-Fi. Moreover, it has an increased Static Random Access Memory (SRAM) of 32 KB that allows better handling of lengthy codes. The updated device is shown in Fig. 2b along with its placement as shown in Fig. 2a. The wearable device consists of different sensors for the heart rate, transthoracic impedance, activity status, and ECG. These sensors include MAX30105 for the heart rate, PMOD Impedance Analyzer based on AD 5933 CE Integrated Circuit (IC) by Analog Devices for transthoracic impedance, ADXL 362 accelerometer for the activity status, and AD 8232 for the ECG [18,[24], [25], [26]]. Two electrode system has been used to measure transthoracic impedance with a start frequency of 80 KHz and stop frequency of 100 KHz, the range of frequencies in which transthoracic impedance response is significant [27]. The transthoracic impedance is measured using the technique called impedance pneumography, where a constant high frequency current generates a potential difference across the thoracic region. The potential difference created is measured in order to measure the impedance of thorax region, known as thoracic impedance. The MAX30105 sensor measures the heart rate based on the photoplethysmography (PPG) principle using LEDs and an optical sensor [28,29]. The MAX30105 measures the change in light absorption using the optical sensor [28]. Moreover, AD 8232 is an ECG signal conditioning block that measures ECG using single lead three electrode system. These three electrodes consist of Right Arm (RA), Left Arm (LA) and Right Leg (RL) and are connected in Einthoven triangle for ECG measurement [25,30]. ADXL 362 is used as a motion activity sensor where it detects whether the subject is active or not active. It has been programmed to report Activity as 1 and Not-Active as 0. ADXL 362 defines activity using two thresholds, acceleration threshold and time threshold where acceleration threshold defines the acceleration above which the subject should be in order to be considered as active and time threshold defines the time for which the subject should be above the acceleration threshold for ADXL 362 to detect the subject as active [26]. In our application, we have chosen the acceleration threshold as 300 codes and the time threshold as 0.1 s such that the ADXL362 is sensitive enough to detect a transitioning of the subject from resting to dynamic condition. The updated device also includes a Storage Disk (SD) card for storing recordings of the parameters [31]. The AD5933 requires to be calibrated with a known value of resistance, in this case 500 Ω, before switching to the unknown thoracic impedance. Unlike the previous device, the updated device includes an automatic switching for calibrating the thoracic impedance sensor. For this purpose, a double pole double throw (DPDT) analog switch, MAX 1680, has been used instead of the previously used manual switch [32]. These sensors were programmed using Arduino IDE, code for which is given in the supplementary information (section 1). Moreover, with the inclusion of the smaller microcontroller, the overall size of the device has decreased from 120.1 × 125 mm^2^ to 126 × 60 mm^2^.

### 2.2 Cloud database

The Arduino MKR 1010 Wi-Fi contains a Wi-Fi module that can be programmed using the built-in library. The device has been programmed to connect and send values to the cloud database. Firebase cloud database by Google has been used for this purpose. There are two types of Firebase cloud database: Realtime database and Firestore database. The Realtime database can be used for storing values in real-time whereas Firestore does not allow real-time storage of the data. In our application, Firestore database stores the data of the registered doctors and patients [33]. The wearable device has been programmed to connect to the Wi-Fi with a username and password authentication. Once the device gets connected to the Wi-Fi, it starts communicating with the sensors to record data. The device sends values of each of the parameters in different nodes of the Firebase Realtime database. The Firebase library of the Arduino allows different functions that can be used to send and receive different types of data into the cloud. The mobile application then reads these values from the Realtime database.

### 2.3 Mobile application

**Fig. 3:**
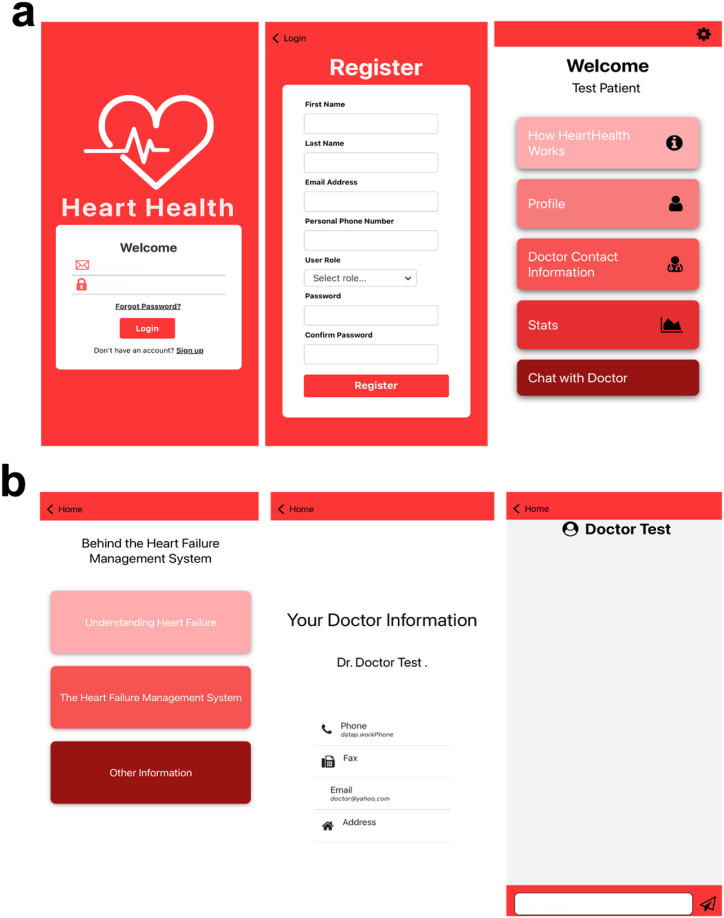
HeartHealth Application User Interface with the different screens a. HeartHealth Login and Registration pages along with User's Main Page b. HeartHealth Mobile Guide about the wearable device and mobile App along with registered doctor and chat box.

A mobile application, HeartHealth as shown in Fig. 3 a,b, developed for iOS and android devices is built using React Native as frontend and Google Firebase's Realtime and Firestore databases as backend with JavaScript as the programming language [[34], [35], [36]]. [34] React Native is a framework that allows classes and functions to develop cross platform mobile applications that can be used on both iOS and Android devices [35]. HeartHealth application is used to view data in real-time and communicate with other users. Users can register as either a doctor or a patient and can view and manage data pertaining to the heart rate, ECG, activity status, and transthoracic impedance. Data is retrieved from and stored in Google's real-time database and Firestore database. The application registers patients and doctors or providers on Firestore database and retrieves their respective vital parameters from the Realtime database. Each doctor and patient are registered with a unique ID in the Firestore database, and the same ID is used to retrieve the parameters from the Realtime database. Fig. 3b shows a graphical user interface of the HeartHealth application. The registration page, as shown in Fig. 3a, stores the user's biometric information along with a unique ID and a role as either doctor or patient. The unique ID is assigned to the patient as associated with the device. The application registers patients under a doctor and doctors are registered with their own unique ID. The application allows the doctor to search for a particular patient registered under that doctor's ID and can monitor his or her parameters. The application is also enabled with a chat feature that allows both doctors and patients to communicate with each other over messaging. The code of the mobile application is provided in the supplementary information (section 2.)

An IoT of the above-mentioned components have been used to develop a telehealth monitoring system and was used by a diverse cohort of subjects to record the parameters. The subsequent paragraphs will discuss the details of the experiment.

### 2.4 Experimentation

The IoT device has been validated with a set of healthy subjects to record the above-mentioned parameters using the proposed telehealth monitoring system. For this purpose, a diverse set of 10 patients with different body types were enrolled with approval by the Institutional Review Board (IRB) at Florida Atlantic University. Each patient's data was recorded for 1 h in different real-time conditions. These conditions include sitting, standing, and walking. Data was recorded for each of the parameters for 20 min in each condition. The overall protocol is summarized in Table 1. The experiment starts with the registration of the patient on the portal where the patient enters their biometric details including gender, weight, and height. Once the registration is complete, the patient can then power the device with the portable battery and the device starts recording the parameters. For the first 20 min, the patient's data is recorded while sitting and then the patient is asked to stand for the next 20 min, and in the end, the patient walks while wearing the device for the last 20 min. This was done to validate the device in real-life conditions and to record the data for an extended period.

**Table 1:** Experiment protocol using the telehealth monitoring system.

Condition	Parameters	Duration (mins)
Sitting	Heart Rate Activity Status Thoracic Impedance ECG	20
Standing	Heart Rate Activity Status Thoracic Impedance ECG	20
Walking	Heart Rate Activity Status Thoracic Impedance ECG	20

## 3 Results

**Fig. 4:**
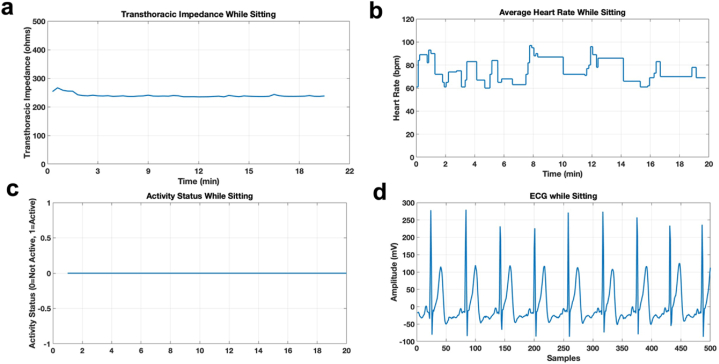
a. Average transthoracic impedance of a subject while sitting b. Average Heart Rate of a subject while sitting. c. Activity status of a subject while sitting d. ECG of a subject while sitting.

The protocol mentioned in Table 1 has been used to run experiments on 10 subjects with different biometric information. The information about the subjects is given in Table 2. There were 6 male and 4 female subjects of varying height and weight with ages between 20 and 33 years. Their vitals were recorded for 1 h and were sent to the mobile application through the database in real-time. Fig. 4 a-d represents one of the subjects’ vitals. Results for the rest of the subjects are provided in the supplementary information section 4. The mobile application shows the vitals of the subject in real-time. The wearable device is programmed to pre-process the parameters before sending them to the mobile application to eliminate any outliers. For this purpose, the values outside the abnormal range of the parameter are filtered. For example, for a healthy subject, as in this case, the heart rate ranges in between 60 and 120 beats per minute (bpm) (in sitting, standing, and walking conditions) therefore any value outside this range due to the uneven pressure on the heart rate sensor, known as noise, is not considered. Similarly, the transthoracic impedance lies in the range of 70–11210 Ω, depending on the subject and the system used for measuring the transthoracic impedance. Therefore, any value less than 70 Ω and greater than 11210 Ω are filtered out because values outside this range are due to either skin resistance or the loose contact of the electrodes with the skin. Each value of a transthoracic impedance is an average of transthoracic impedances obtained at the significant frequency range of 80–100 KHz.

**Table 2:** Subjects with their biometric information.

Subject	Gender	Age	Height (cm)	Weight (lbs)
1	M	27	165	135
2	M	33	189	187
3	M	30	160	132
4	M	29	168	220
5	M	31	168	187
6	M	28	168	176
7	F	28	162.56	97
8	F	26	161.29	125.6
9	F	28	131	143.5
10	F	27	160.02	176.37

**Fig. 5:**
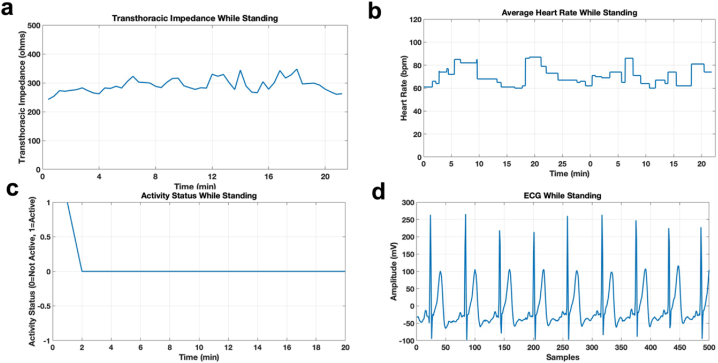
a. Average transthoracic impedance of a subject while standing b. Average Heart Rate of a subject while standing. c. Activity status of a subject while standing d. ECG of a subject while standing.

**Fig. 6:**
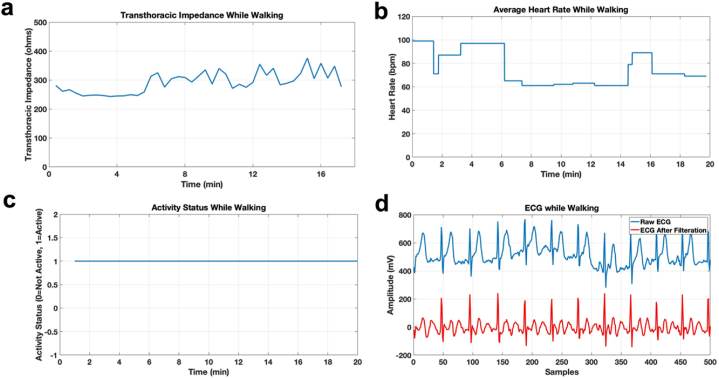
a. Average transthoracic impedance of a subject while walking b. Average Heart Rate of a subject while walking. c. Activity status of a subject while walking d. ECG of a subject while walking.

Fig. 4, Fig. 5, Fig. 6 show the vitals of the subject in different conditions. Fig. 4 shows the vitals while sitting with activity status being 0 at all times as shown in Fig. 4c. Fig. 5a–d shows the vitals in the standing condition and Fig. 6a–d shows the vitals in the walking condition. It can be seen in Fig. 5c that while standing, the activity status was active (1) for the instance when the subject stood up whereas for 6c the status is active for all instances while the subject is walking. The transthoracic impedance and heart rate are in the normal range with slight variations as shown in Fig. 4a and b, Fig. 5a and b and Fig. 6a and b in three different conditions. Moreover, for the ECG it was clean and stable while sitting and standing however it was noisy while walking due to motion artifact, as can be seen in Fig. 4, Fig. 5, Fig. 6d. Fig. 6d shows the raw ECG of a subject while walking as well as a filtered ECG. The ECG from the belt in walking condition was post-processed in MATLAB. The low-frequency motion artifact was removed using a high pass filter with a cut-off frequency of 10Hz, sampled at 200Hz. Moreover, for the purpose of comparison, DC offset from all ECGs was removed in all conditions.

**Fig. 7:**
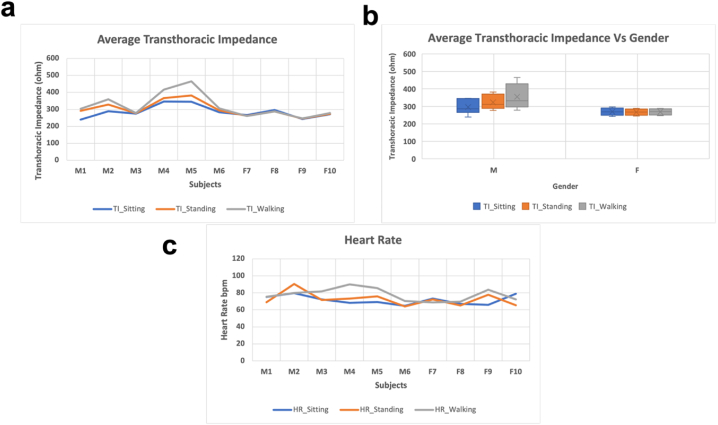
a. Average transthoracic impedance of different subjects in different conditions: sitting, standing, and walking. b. Average transthoracic impedance for different genders. c. Average heart rate of subjects in different conditions.

Table 3 shows the average values of the vital parameters for different subjects whereas these results are graphed in Fig. 7. Fig. 7a represents the average value of transthoracic impedance in different conditions for all subjects. It can be seen from Fig. 7b that on average males have higher transthoracic impedance than females. This is because of the difference in the average body to mass index (BMI) in male and female volunteers. An increased BMI increases the resistivity of the region and hence the resistance of the region, in this case thoracic region [37]. This can be validated from Fig. 7a where subject 4 and 5 (males) have comparatively higher transthoracic impedance than the rest of the volunteers and can be seen in Table 2 that they also have higher BMI than the rest of the volunteers (eq. (1) and eq. (2)). Similarly, Fig. 7c represents the average heart rate values in different conditions for different subjects. Expectedly the average heart rate for most of the patients while walking is higher than the resting heart rate in sitting and standing.

(1) $$BMI=\frac{Weight}{Height^{2}}$$

(2) $$R=\frac{pL}{A}$$

**Table 3:** Subjects with their average values in different conditions.

Subject	TI_Sitting	TI_Standing	TI_Walking	HR_Sitting	HR_Standing	HR_Walking	AS_Sitting	AS_Standing	AS_Walking
1	239.53	291.66	303.47	75.1355	69.00	75.51	0	0	1
2	288.91	329.17	359.85	79.6581	90.43	80.00	0	0	1
3	274.54	277.55	278.25	72.2823	71.74	81.94	0	0	1
4	345.87	366.26	416.69	68.3339	73.25	89.83	0	0	1
5	345.47	381.81	465.23	69.2979	75.89	85.55	0	0	1
6	283.64	294.18	306.18	64.7256	64.00	70.49	0	0	1
7	267.12	261.71	260.53	73.2672	72.41	68.82	0	0	1
8	297.05	288.76	288.54	67.1399	65.13	69.74	0	0	1
9	242.87	244.92	247.19	65.9978	77.69	83.66	0	0	1
10	271.02	274.01	278.99	78.8822	65.36	72.43	0	0	1

## 4 Discussion

A telehealth system for the continuous and real-time monitoring of the parameters of HF has been developed. The system is based on three modules: the wearable device, the cloud database, and the mobile application. The wearable device is an IoT device that integrates different sensors for measuring different parameters namely transthoracic impedance, ECG, heart rate, and activity status. These parameters are stored in a SD card and on the cloud database. The same parameters can also be visualized on the mobile application. The telehealth system provides an effective means to monitor HF vital parameters with a feature to communicate with the medical provider. The system has been tested in different real-time conditions including sitting, standing, and walking for different subjects. In the results shown above, the telehealth system successfully kept track of all the parameters. It was observed that the transthoracic impedance varies with the BMI: greater the BMI, greater the resistivity of the region and hence greater the impedance will be. Moreover, it has also been observed that on average for both genders, the resting heart rate was lower than the heart rate while walking. Considering the progression of HF, a telehealth monitoring system like this is indeed timely. There are currently some wearables available for monitoring general vitals including heart rate, oxygen saturation, and respiration rate. For example, Savvy is one such device that measures ECG and shares the ECG with the medical provider [38]. Similarly, VitalPatch by VitalConnect is another wearable device that measures ECG, heart rate, respiration rate, R–R interval, activity status and posture detection [39].

Most of the telehealth monitoring devices have the limitation of the power supply to keep track of the vitals for extended periods [15,40]. The proposed device consumes around 105 mA and hence can be powered with a small 1100 mAh device for 10.5 h. Telehealth monitoring systems also have the limitation of data privacy and protection. Personal health information is valuable and therefore telehealth monitoring systems need to be protected [[41], [42], [43]]. For this purpose, our proposed device is specified with a subject's ID and is also protected with the user-defined password on the mobile application. Moreover, as part of our future direction, we intend to further reduce the size of the printed circuit board (PCB) by making a system-on-chip device along with the validation of our device on actual HF patients with implanted ICDs.

## 5 Conclusion

A telehealth monitoring system has been developed that allows the continuous and real-time monitoring of parameters that are significant for HF. These parameters include transthoracic impedance, ECG, heart rate, and activity status. These parameters are stored on the cloud database as well on the SD card and can also be visualized on the mobile application. The mobile application allows the registration of both the doctor and the patient with a feature to communicate with each other over chat messages. The system has been validated with different subjects under different conditions including sitting, standing, and walking. The device connects with Wi-Fi and shares the processed data over the cloud database in the respective repository of the subject, based on his or her ID. The mobile application is connected to the database and can be used to visualize when logged in with the respective username and password. This non-invasive integrated telehealth device can be a useful tool to care for the 32 million patients worldwide who have HF with preserved ejection fraction.

## Data availability statement

The data described in this manuscript is openly available and is provided in the supporting document. It can also be found here: https://github.com/ShkhAsher/A-telehealth-system-for-the-monitoring-of-parameters-related-to-heart-failure..git.

## CRediT authorship contribution statement

**Sheikh MA. Iqbal:** Experimentation, Methodology, Conceptualization, Writing – review & editing. **Mary Ann Leavitt:** Writing – review & editing, Methodology, Conceptualization. **Guerline Pedilus:** Software, Conceptualization. **Imadeldin Mahgoub:** Writing – review & editing, Methodology, Conceptualization. **Waseem Asghar:** Writing – review & editing, Validation, Supervision, Methodology, Conceptualization.

## Declaration of competing interest

The authors declare the following financial interests/personal relationships which may be considered as potential competing interests: Waseem Asghar reports financial support was provided by 10.13039/100000002National Institutes of Health, Award no. R61AI154643. Waseem Asghar reports financial support was provided by 10.13039/100000001National Science Foundation, CAREER 1942487. If there are other authors, they declare that they have no known competing financial interests or personal relationships that could have appeared to influence the work reported in this paper.
