# Supplementary material for: A wearable telehealth system for the monitoring of parameters related to heart failure
Source: Heliyon. 2024 Feb 22;10(5):e26841. doi: 10.1016/j.heliyon.2024.e26841 (PMC10909713; doi:10.1016/j.heliyon.2024.e26841)
Supplement: Multimedia component 1 [file mmc1.docx]

**A wearable telehealth system for the monitoring of parameters related to heart failure.**

1. **Arduino Code:**

The code used for the wearable device is based on the Arduino and can be accessed here:

<https://github.com/ShkhAsher/A-telehealth-system-for-the-monitoring-of-parameters-related-to-heart-failure..git>

1. **HeartHealth Mobile App Code:**

The HeartHealth application has been developed using React Native as the frontend, JavaScript as the programming language and firebase as its database. For this different screen of the mobile application has been developed using React Native’s cross platform classes and functions that allows the development of the application that can be used by both iOS and Android. The code can be found in the following repository:

<https://github.com/ShkhAsher/A-telehealth-system-for-the-monitoring-of-parameters-related-to-heart-failure./tree/e3567595e8cc30a623e02832d7b555f029f5177b/HeartHealth>

1. **Firebase Database:**

**
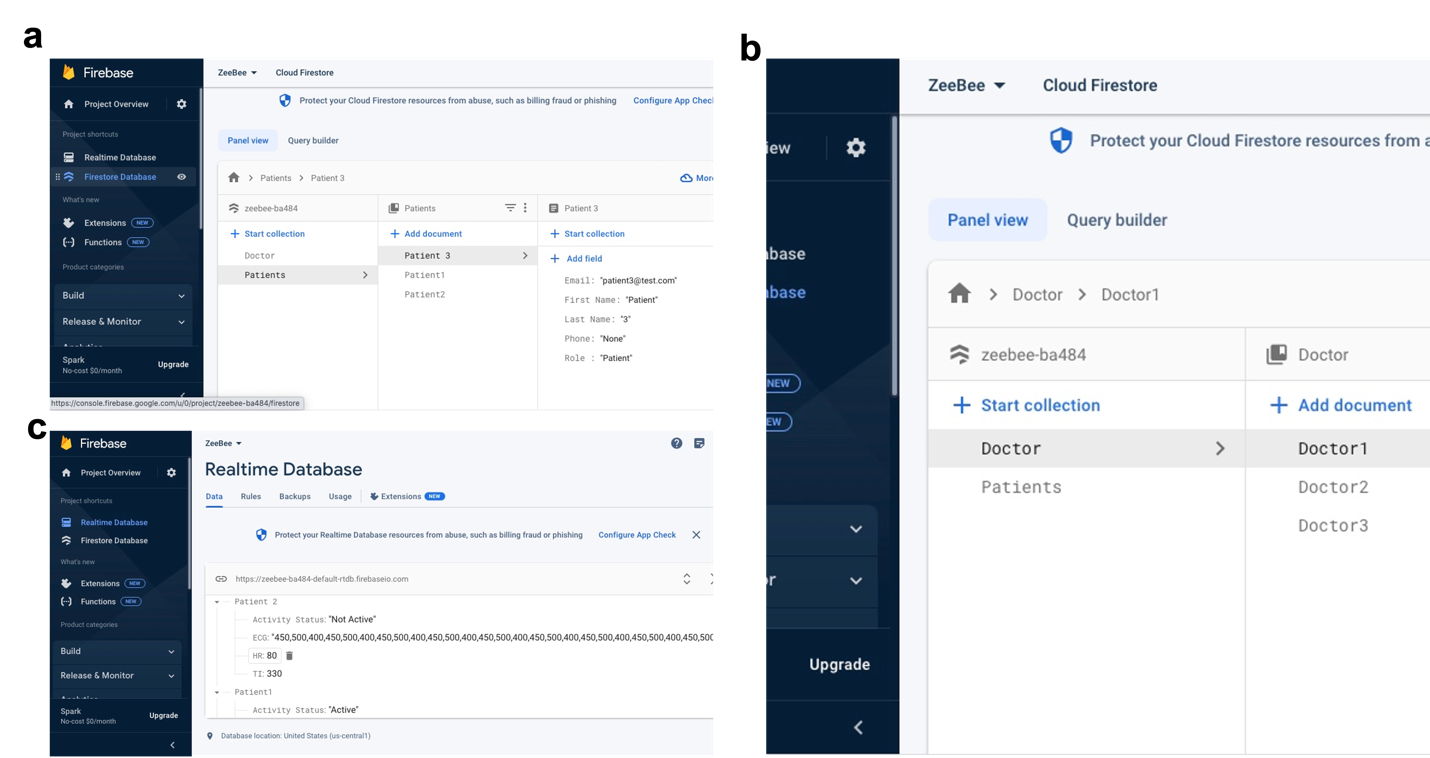
**

**Fig.1** Google Firebase Database a. Firestore Database with Nodes for Doctors b. Firestore Database with Nodes for Patients c. Realtime Database for storing parameters from the patients.

1. **Patient Data:**

**Patient1:**

**
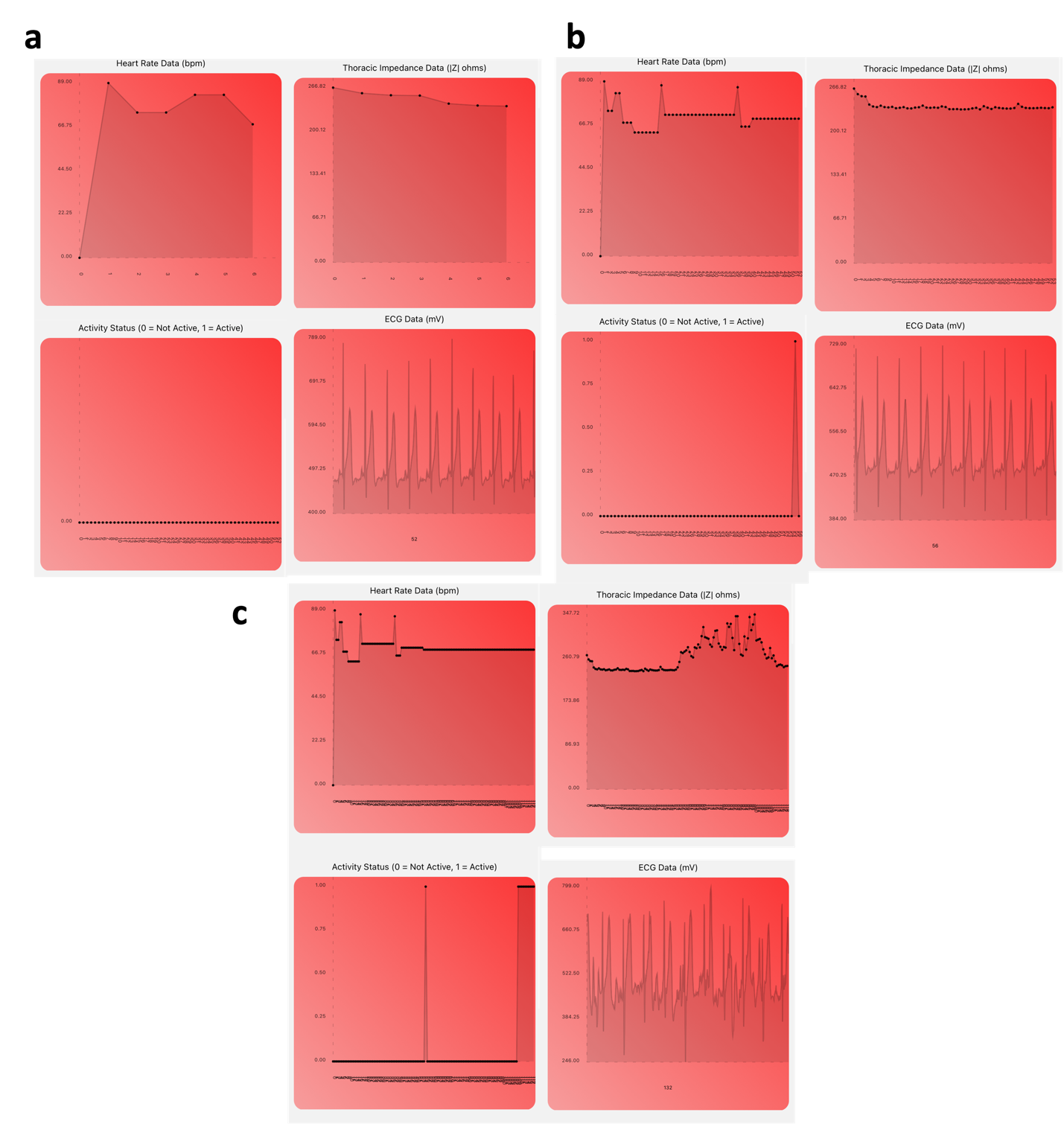
**

a Patient 1 parameters while sitting b. Patient 1 parameters while standing c. Patient 1 parameters while walking.

**Patient 2:**


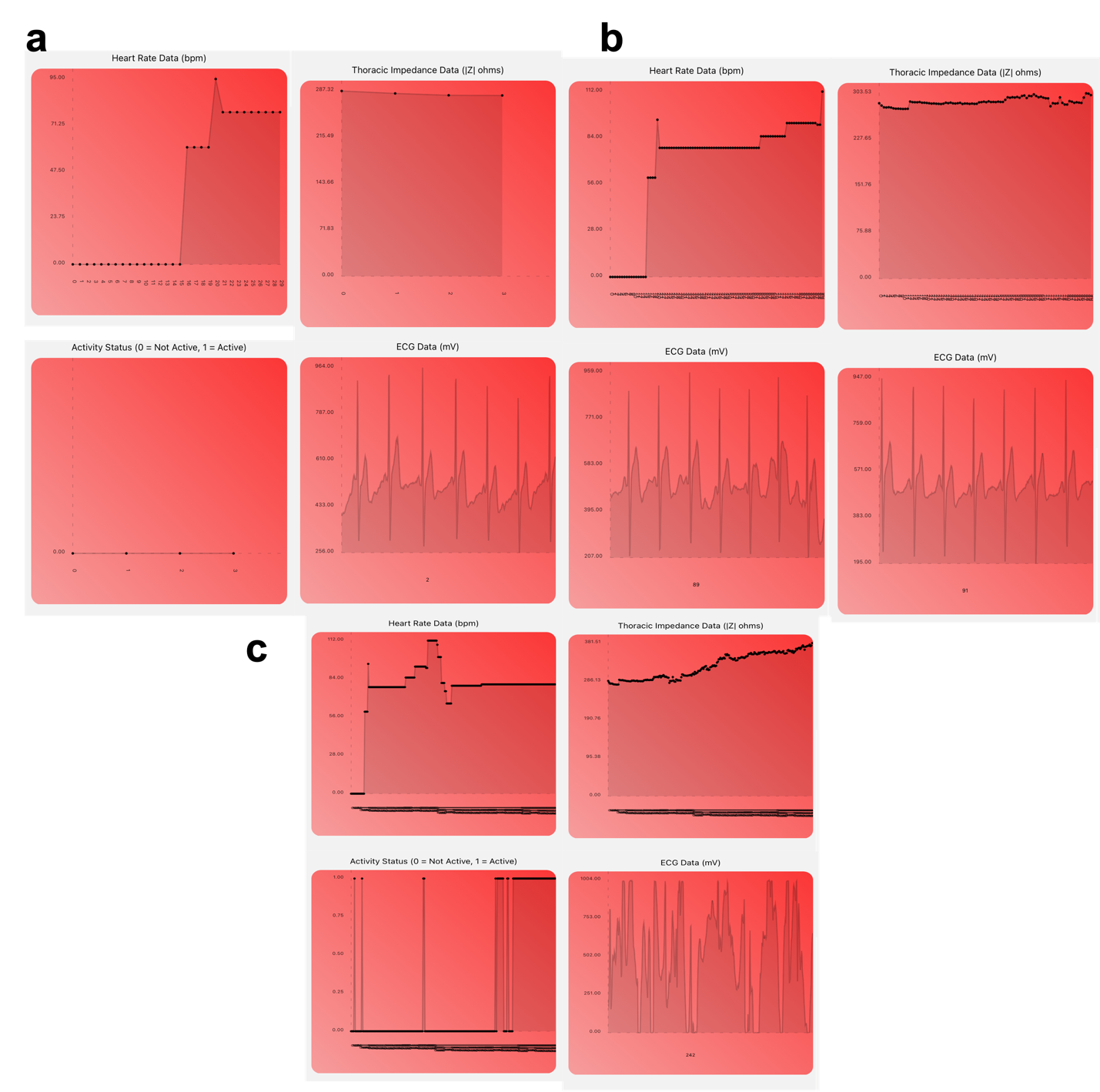


a Patient 2 parameters while sitting b. Patient 2 parameters while standing c. Patient 2 parameters while walking.

**Patient 3:**


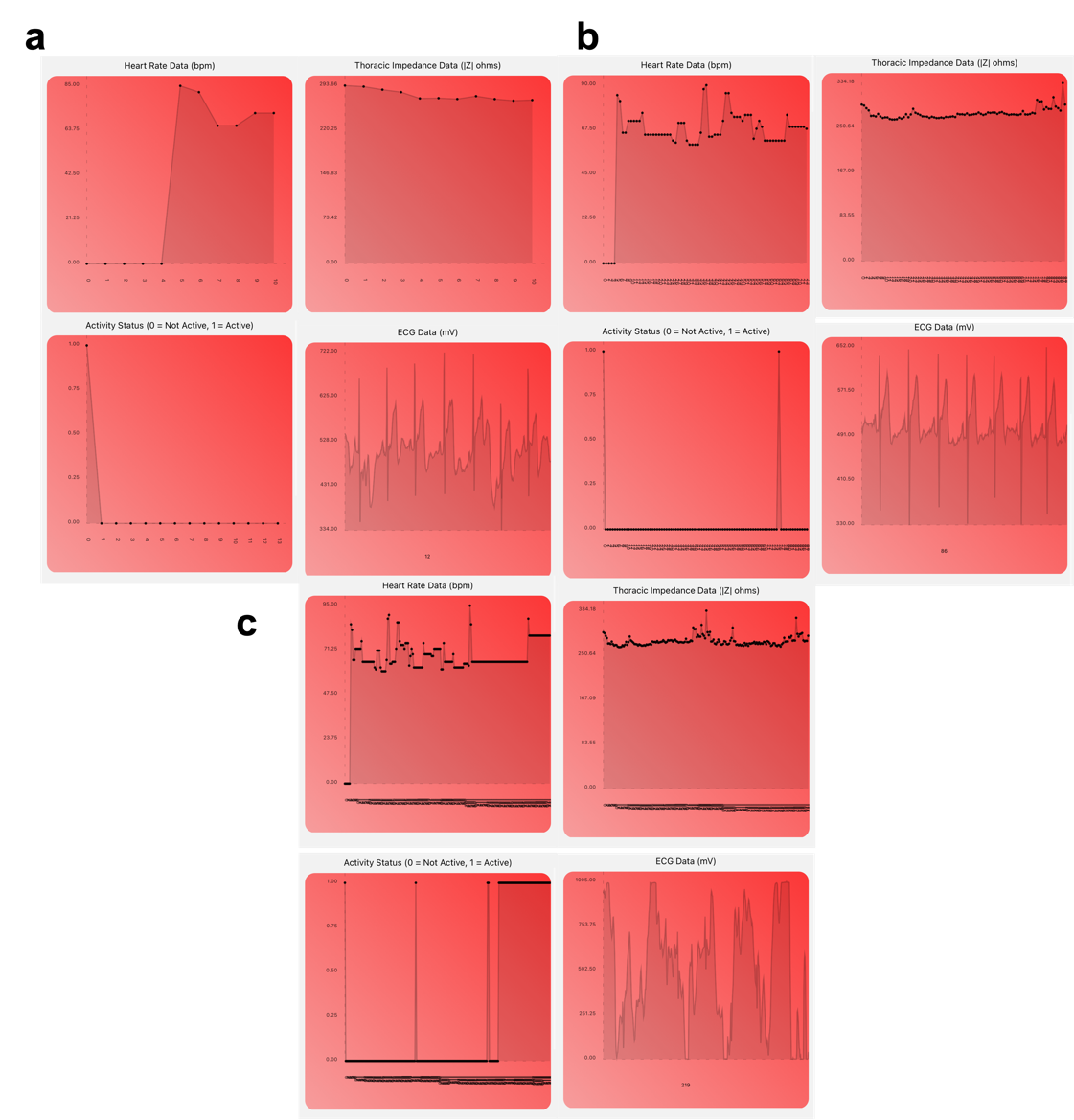


a Patient 3 parameters while sitting b. Patient 3 parameters while standing c. Patient 3 parameters while walking.

**Patient 4:**


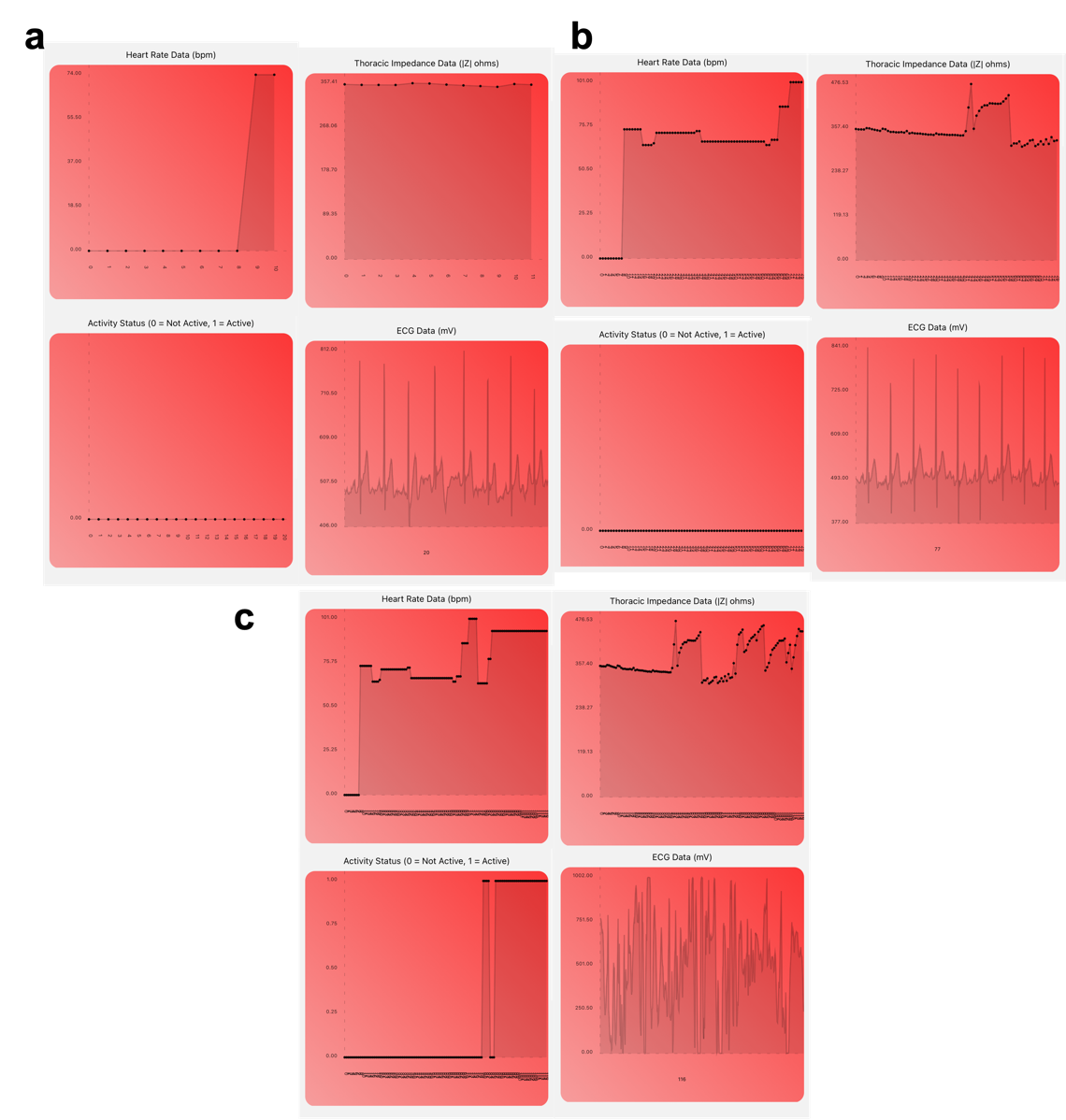


a Patient 4 parameters while sitting b. Patient 4 parameters while standing c. Patient 4 parameters while walking.

**Patient 5:**


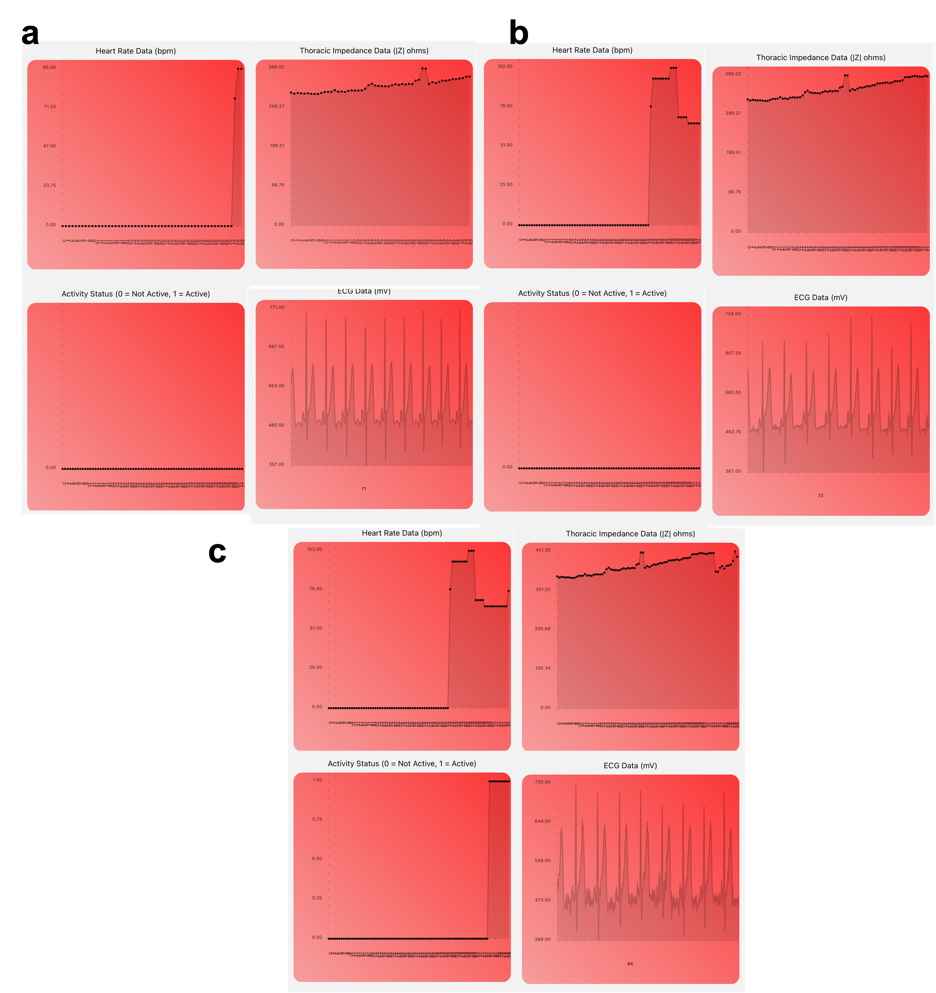


a Patient 5 parameters while sitting b. Patient 5 parameters while standing c. Patient 5 parameters while walking.

**Patient 6:**


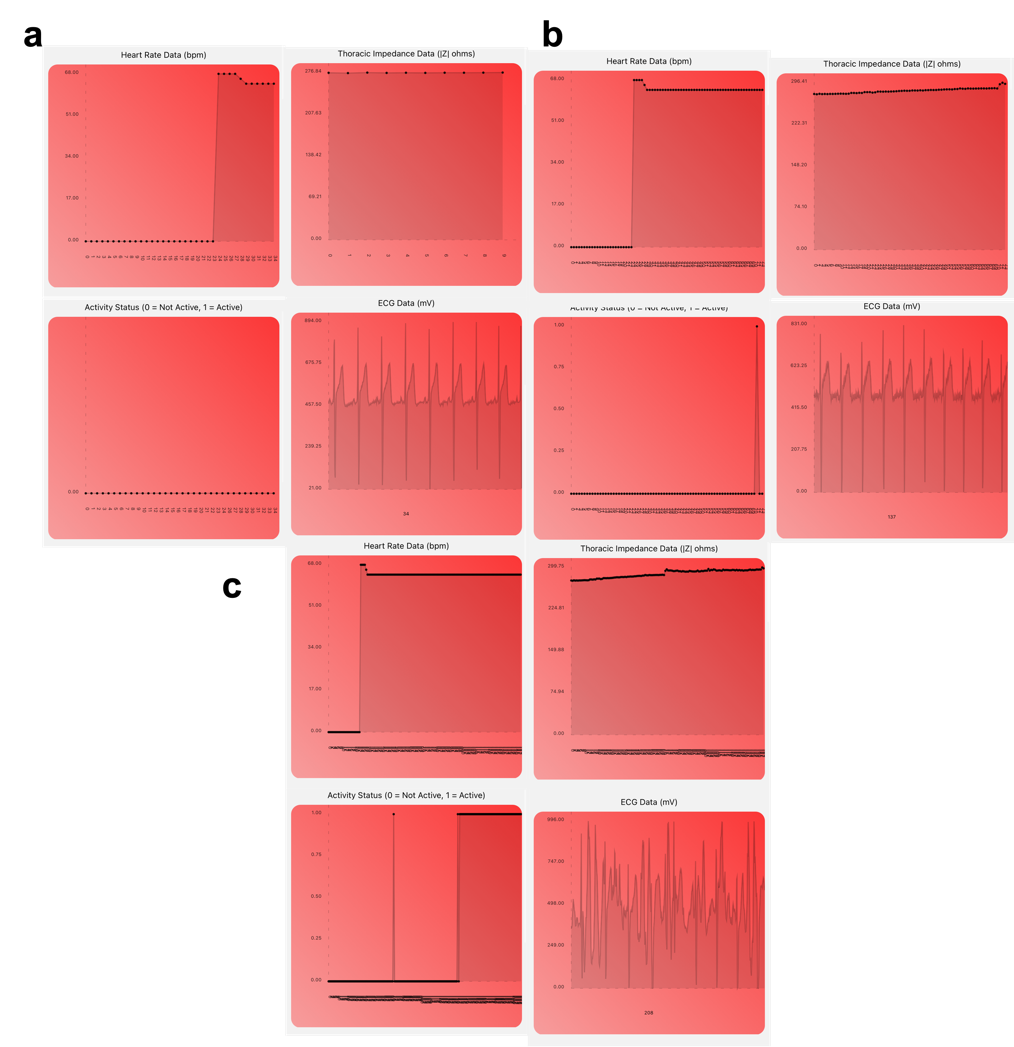


a Patient 6 parameters while sitting b. Patient 6 parameters while standing c. Patient 6 parameters while walking.

**Patient 7:**


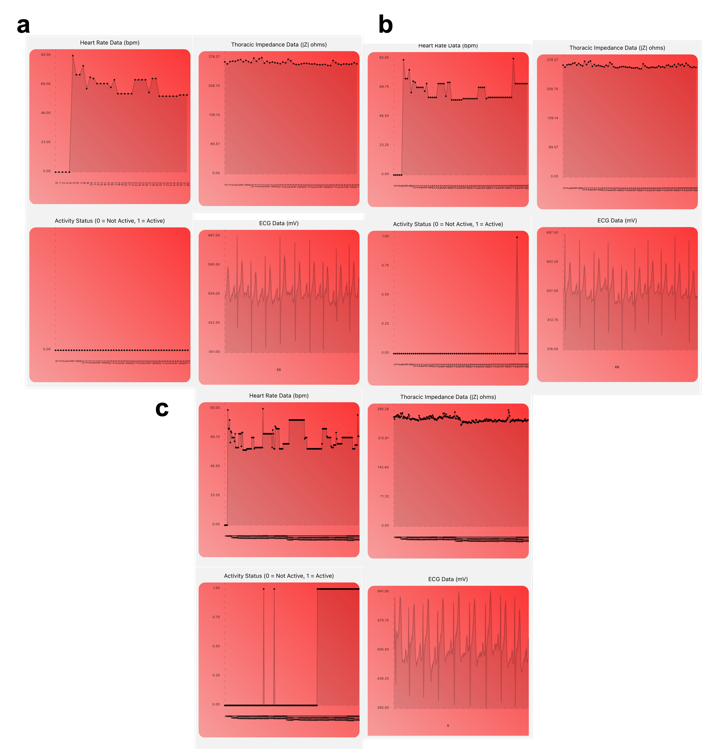


a Patient 7 parameters while sitting b. Patient 7 parameters while standing c. Patient 7 parameters while walking.

**Patient 8:**


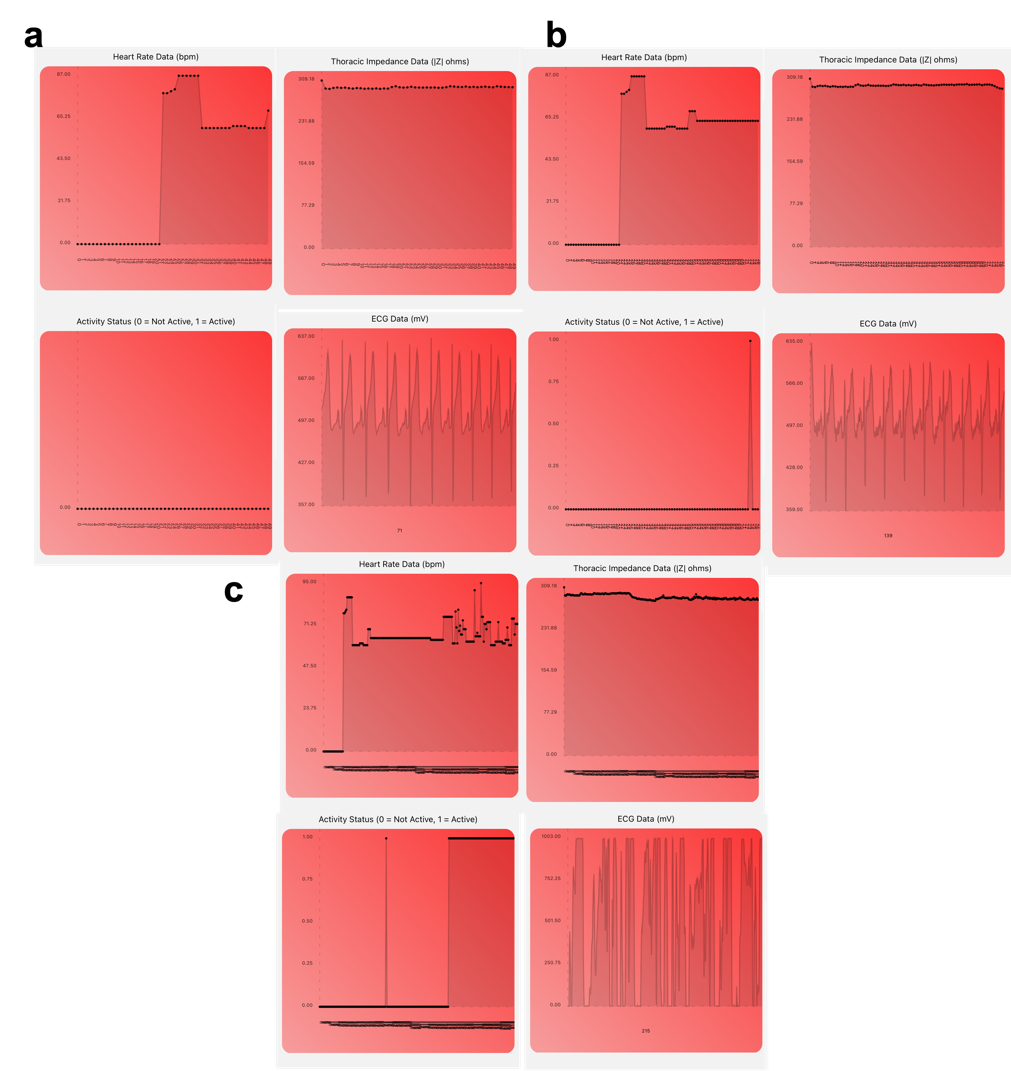


a Patient 8 parameters while sitting b. Patient 8 parameters while standing c. Patient 8 parameters while walking.

**Patient 9:**


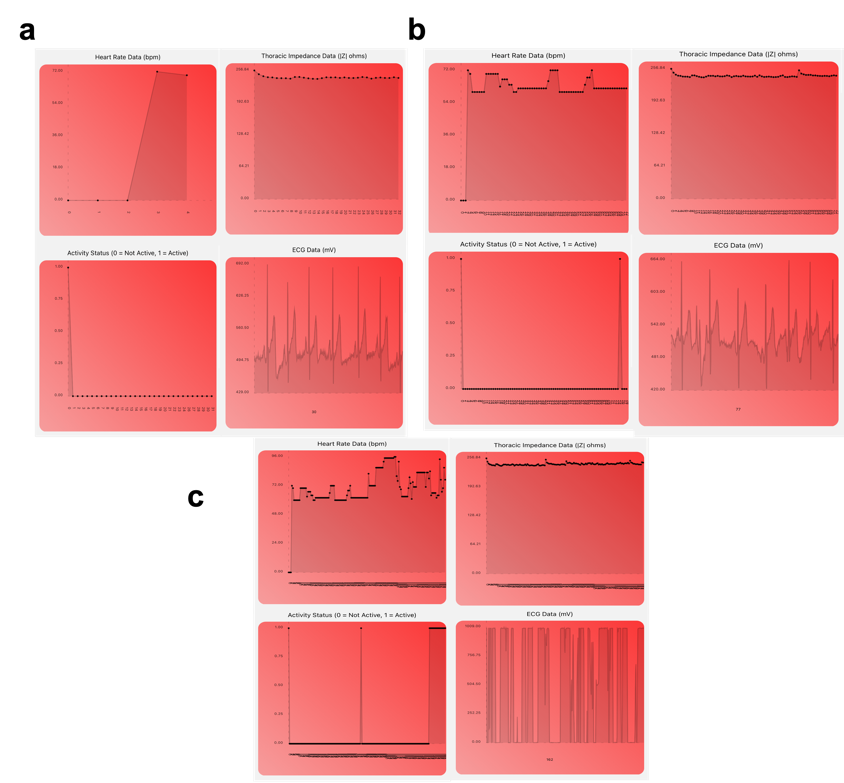


a Patient 9 parameters while sitting b. Patient 9 parameters while standing c. Patient 9 parameters while walking.

**Patient 10:**


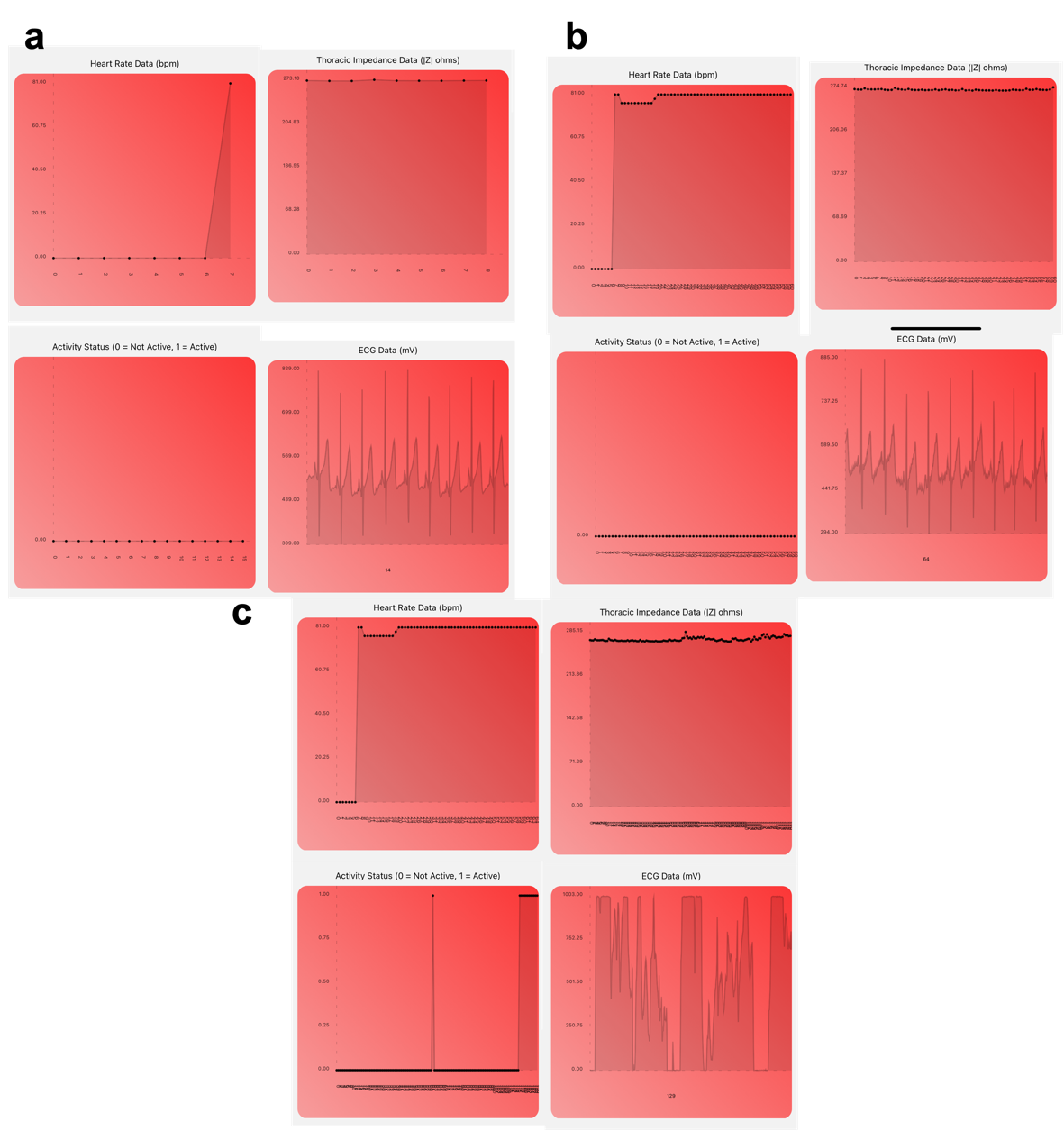


a Patient 10 parameters while sitting b. Patient 10 parameters while standing c. Patient 10 parameters while walking.
